# Effects of Nutrients on Platelet Function: A Modifiable Link between Metabolic Syndrome and Neurodegeneration?

**DOI:** 10.3390/biom11101455

**Published:** 2021-10-04

**Authors:** Ilse A. C. Arnoldussen, Renger F. Witkamp

**Affiliations:** 1 Human Nutrition & Health, Department of Agrotechnology and Food Sciences, Wageningen University & Research, 6708 WE Wageningen, The Netherlands; Renger.Witkamp@wur.nl; 2 Department of Medical Imaging, Anatomy, Radboud University Medical Center, Donders Institute for Brain, Cognition and Behaviour, 6525 EZ Nijmegen, The Netherlands

**Keywords:** platelets, metabolic syndrome, neurodegeneration, nutrients

## Abstract

Metabolic syndrome increases the risk of vascular dementia and other neurodegenerative disorders. Recent studies underline that platelets play an important role in linking peripheral with central metabolic and inflammatory mechanisms. In this narrative review, we address the activation of platelets in metabolic syndrome, their effects on neuronal processes and the role of the mediators (e.g., serotonin, platelet-derived growth factor). Emerging evidence shows that nutritional compounds and their metabolites modulate these interactions—specifically, long chain fatty acids, endocannabinoids and phenolic compounds. We reviewed the role of activated platelets in neurovascular processes and nutritional compounds in platelet activation.

## 1. Introduction

Platelets, the smallest anucleate cells in our blood, can rapidly respond to environmental changes and are best known for their essential contribution in hemostasis, thrombosis and wound healing [1,2]. At the same time, platelet hyperactivity is found in metabolic syndrome (MetS), a cluster of conditions related to abdominal obesity, reduced insulin sensitivity and cardiovascular abnormalities [3,4]. Interestingly, several dietary bioactive compounds are known, including n-3 long chain polyunsaturated fatty acids (LC-PUFAs), vitamins and polyphenols that not only play crucial roles in the prevention and development of MetS, but are also involved in maintaining normal platelet function. Next to this, there is increasing scientific evidence for a convergence of both fields of interest, MetS and platelets, when it comes to Alzheimer’s disease (AD) and vascular dementia. On the one hand, it is a well-known observation that MetS increases the risk of progression from mild cognitive impairment to dementia and the incidence of vascular dementia and AD [5,6]. On the other hand, emerging data underline that platelets may play possibly crucial roles in neurovascular signaling and blood–brain interactions and thereby neurodegenerative disorders such as AD, as was recently reviewed by Leiter et al. [7,8].

This raises the question of which role platelets could play in the interactions between peripheral metabolic dysregulation, inflammation and neurodegenerative processes, and whether dietary active compounds could affect these processes. This is underlined by recent insights demonstrating that platelets can take up, transport and secrete various mediators that are of relevance for both MetS and brain neuronal and immunological functions, including the functionality of the blood-brain-barrier [7,8]. Additionally, platelets, because of their size, can circulate in the capillaries (diameter ranges between 3.0 and 7.0 µm [9]) of the brain. Moreover, microvesicles secreted by platelets can cross the blood–brain barrier (BBB). In more detail, human platelets have a diameter ranging between 1.5 and 3.0 µm and the diameter of platelet secreted microvesicles can either range between 80 and 200 nm or between 400 and 600 nm [10], whereas the diameter of T-cells ranges between 5.0 and 7.0 µm and that of red blood cells is around 7.8 µm.

Therefore, the aim of this narrative review is to summarize and evaluate the available evidence for the role of nutritional compounds with respect to platelet function, focusing on their possible bridging functions between MetS and neurovascular processes. To this end, we first discuss the activation of platelets in MetS, and next the contribution of activated platelets in neurovascular and neurological processes. Thirdly, we review nutritional compounds modulating the activation of platelets. With this review, we aim to contribute to the understanding of platelets and their effects in brain function in MetS and to provide possible new directions for diet-based therapeutic interventions.

## 2. Platelet Activation in Metabolic Syndrome

**Figure 1:**
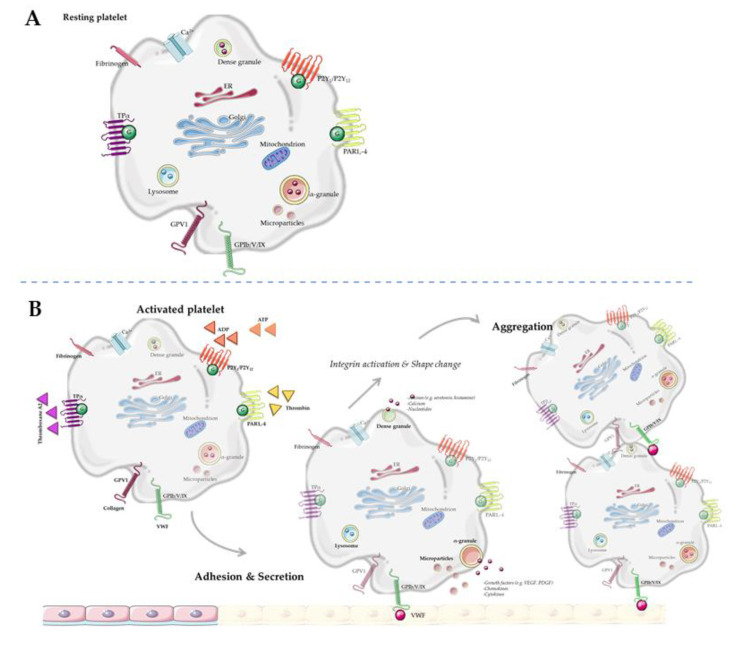
Schematic representation of platelet activation. (**A**) Resting platelet. (**B**) Platelet activation, adhesion and aggregation.

Platelets are derived from megakaryocytes in the bone marrow, and are equipped with various secretory vesicles, messenger ribonucleic acid (mRNA) and mitochondria [11]. Multiple metabolic enzymes are stored in and released upon activation from these secretory vesicles: lysosomes, dense granules, α-granules, exosomes and microvesicles (Figure 1). In general, lysosomes mainly store clearing factors such as acid proteases and glycohydrolases. Dense granules contain pro-aggregating factors such as nucleotides (adenosine triphosphate (ATP), adenosine diphosphate (ADP)), amines (serotonin, histamine, γ-aminobutyric acid (GABA), glutamate, epinephrine, dopamine, and histamine) and calcium [8,12]. α-Granules contain adhesion and repairing factors such as growth factors, chemokines, cytokines, protease inhibitors and adhesive glycoproteins, as reviewed in detail by Rendu et al. [12]. Platelet microvesicles are derived from multivesicular bodies and contain multiple bioactive molecules, RNA and proteins, mainly but not exclusively from α-granules [13,14,15,16]. The ability to change in shape and the high turnover rate of platelets (8–12 days [17]) underline that they are able to respond to changing environmental conditions. Interestingly, the content of platelet vesicles depends on specific environmental factors which activate the parent platelet [18,19,20,21]. For example, at infection sides, platelet microvesicles contain specific factors to recruit leukocytes [21]. Next to this, molecules in α-granules can be, in addition to being inherited from the parental platelet, collected via endocytosis [22,23].

Metabolic syndrome is defined as a cluster of metabolic risk factors, which include obesity, hypertriglyceridemia, high low-density lipoprotein levels in blood, hypertension, and hyperglycaemia with insulin resistance (reviewed by [24]). Interestingly, MetS has been associated with platelet hyperactivity [25,26,27,28]. Zaccardi et al. concluded in their meta-analysis that particularly individuals with type two diabetes mellitus (T2DM) showed increased platelet activation, as parameters for platelet activation such as mean platelet volume and platelet distribution width were higher in these individuals [28]. However, more recent articles, not included in the meta-analysis of Zaccardi et al., suggested that increased levels of platelet activation are typical for persons with MetS in a general sense, not being specifically limited to T2DM [29,30,31]. For example, in a recent study involving 18 individuals with MetS, an enhanced platelet activation state was found compared to that in the age-matched control group [29]. Similarly, in a study with obese adolescents, both inflammatory state and platelet activation were increased as compared to the control population [23]. Finally, a recent study showed that individuals diagnosed with MetS and coronary artery disease had a higher MPV than patients without MetS [30]. However, MetS did not emerge as an independent predictor of higher MPV values [30]. Together, these studies support the idea that platelet activation is associated with MetS, although it remains difficult to identify the main underlying actor(s). Notably, women often have a higher platelet count and enhanced platelet activation compared to men, which is plausibly caused by a higher expression of surface receptors, whereas ADP-dependent platelet reactivity has been found not to differ between men and women [32]. A study by Zhao et al. reported that increased mean platelet volume (MPV) is inversely associated with the risk of developing MetS in Chinese women, but not in men [31]. These findings might indicate sex-specific differences in platelet activation in MetS, and underline the importance of taking sex differences into account in future research in this field.

The most obvious processes that contribute to platelet activation in MetS are considered to be hyperglycemia and dyslipidemia (reviewed by [27]). For example, high glucose levels were found to enhance platelet reactivity in human blood through elevated osmolality, which occurred by means of superoxide anion production [33]. It was also found that high glucose levels induced platelet secretion via adenosine diphosphate (ADP)-induced platelet P-selectin expression, and moreover increased thrombin receptor-activating peptide (TRAP)-induced platelet P-selectin expression and fibrinogen binding by enhancing protein kinase C (PKC)-signaling [33]. Another component of MetS, dyslipidemia, could also be linked to induced platelet activation, as shown by the effects of high levels of low-density lipoprotein (LDL). Here, activation of platelets can be induced via multiple mechanisms, for instance via a reduction in the intracellular pH (pHi) of platelets mediated by LDL. Specifically, LDL was found to inhibit the platelet antiport Na^+^/H^+^, thereby reducing platelet pHi, which in turn caused increased platelet reactivity [34]. A second mechanism of platelet activation can occur via oxidized LDL. Oxidation of LDL is catalyzed by metal ions (e.g., copper, iron), oxidizing enzymes (e.g., myeloperoxidase and other peroxidases, lipoxygenase, xanthine oxidase, nicotinamide adenine dinucleotide phosphate (NADPH) oxidase and other superoxide-generating enzymes), or occur via the generation of peroxynitrite, nitric oxide and thiols (reviewed by [35]). Interestingly, it can also be caused by platelets themselves [36]. In more detail, Carnevale et al. reported that when exposed to native LDL, activated platelets generated oxidized LDL, which in turn served to further propagate platelet activation [36]. NADPH oxidase 2-derived reactive oxygen species (ROS) have a central role in both events, as on one hand they contributed to LDL oxidation, while on the other hand they served as intra-platelet signaling mediators to activate platelets by oxidized LDL [36]. Additionally, dyslipidemia is associated with enhanced oxidant stress and synthesis of oxidized lipids, and specifically oxidized choline glycerophospholipids induce platelet aggregation via CD36 [37]. A third mechanism involving circulating LDL occurs through its glycation, which was found to result in an increased intracellular calcium concentration and increased cytosolic calcium concentrations in platelets, thus stimulating platelet nitric oxide synthase (NOS) activity [38]. Glycated LDL particles are more susceptible to oxidative changes than native LDL [39], thereby increasing their potency in order to activate platelets. Additionally, glycoxidized LDL increased the phosphorylation of platelet p38 mitogen-activated protein kinase (MAPK), as well as the concentration of thromboxane B2 in individuals with T2DM [40]. Yet another proposed link comes from the observation that LDL from individuals with MetS and T2DM can activate platelets and collagen-induced platelet aggregation via the platelet arachidonic signaling cascade [41]. Platelet arachidonic acid signaling cascade was activated by LDL via the phosphorylation of p38 MAPK, cytosolic phospholipase A2 and increased thromboxane B2 formation [41,42,43]. Finally, it is known that MetS, obesity and T2DM are strongly associated with the development of low-grade systemic inflammation [44,45,46]. Low-grade systemic inflammation can induce platelet hyperactivity via an increased expression of soluble P-selectin, enhanced levels of pro-inflammatory molecules such as prothrombin molecule cluster of differentiation-40, C-reactive protein (CRP) and pro-inflammatory cytokines, interleukin-6 and tumor-necrosis-factor-alpha (TNF-α) (reviewed in [26,27]).

**Figure 2:**
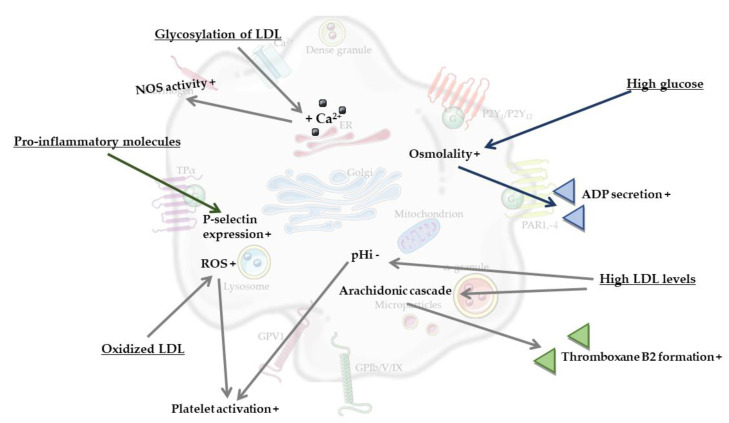
Mechanisms by which platelets can be activated in metabolic syndrome.

Taken together, MetS appears to be associated with an increased state of platelet activation, most probably caused by overlapping features such as hyperglycaemia, dyslipidaemia, and low-grade systemic inflammation. These components of the MetS increase platelet osmolality and calcium concentration, activation of the platelet arachidonic signaling cascade, oxidation and glycation of LDL and levels of pro-inflammatory molecules, which all increase platelet activation (Figure 2). Clearly, MetS is also associated with increased risks of cardiovascular disease, vascular dementia and Alzheimer’s disease (AD) [5,6,47]. Combined with the changes in platelet function during MetS described above, this underlines the hypothesis that a chronically elevated level of platelet activation may play a bridging role between hyperlipidaemia, hyperglycaemia, low-grade systemic inflammation and neuroinflammation, structural brain changes and even neurodegeneration.

## 3. Platelets and Neurological Processes

Metabolic syndrome has been associated with both increased levels of activated platelets and with dementia, in particular vascular dementia and AD [5,6]. Activated platelets could contribute to the pathological neurodegenerative processes in MetS, most probably via modulation of the crosstalk between neurons and the vasculature. Interestingly, alterations in platelet function have been found in mild cognitive impairment (MCI) and AD [47,48]. In addition, higher numbers of platelets were located within the brain parenchyma in an AD mouse model. These platelets were in close contact with astrocytes and were activated as manifested by the expression of the platelet activation marker, P-selectin [49]. Interestingly, when these platelets were isolated and transferred into the brain of wildtype mice, neuroinflammatory processes and vessel damage were induced [50,51]. Thus, activated platelets might modulate neurological processes in metabolic and vascular disorders. In the following subsections, we review plausible mechanisms by which activated platelets could negatively affect the permeability of the blood–brain barrier (BBB), neurogenesis, myelinization and neuroinflammation.

### 3.1. Platelets and BBB Permeability

The BBB, a structure designed to allow selective uptake of nutrients and restricting entry of toxins, immune cells and pathogens to the brain, is essential for proper neuronal function. Recently reviewed by Van Dyken et al., local and low-grade systemic inflammation induced by MetS can cause functional deterioration of the BBB as manifested by decreased removal of waste, increased permeability and infiltration of immune cells [52]. Functional decline of the BBB can also lead to disruption of glial and neuronal cells, causing hormonal dysregulation, increased immune sensitivity and cognitive impairment depending on the affected brain regions [52]. Activated platelets can release a variety of growth factors which can modulate the abovementioned processes like increased BBB permeability and infiltration of immune cells. Examples of these growth factors include platelet-derived growth factor (PDGF), vascular endothelial growth factor (VEGF), brain-derived neurotrophic factor, platelet factor 4 (PF4), transforming growth factor-ß, fibroblast growth factor (FGF), and insulin-like growth factor-1, as has been extensively reviewed by Burnouf et al. and Golebiewska et al. [53,54]. VEGF released by platelets stimulates angiogenesis resulting in immature, unstable vessels, and increases BBB permeability [52]. From a mechanistical perspective, VEGF has been shown to increase the BBB’s permeability via alterations in the expression and distribution of tight junction proteins [52,55]. On the other hand, platelets are a major source of platelet-derived growth factor B (PDGFB), and the release of platelet-derived PDGFB could promote and maintain vascular integrity via recruitment of pericytes [56]. In addition, the dense granules in the platelets abundantly store serotonin and histamine, and both factors have shown to be vasoconstrictive. Interestingly, obesity, often associated with MetS, has been associated with decreased levels of cerebral perfusion [57]. In a rodent model, the administration of serotonin via an external carotid catheter, when the BBB was intact, resulted in a reduction in cerebral blood flow in the caudate nucleus [58]. However, following BBB disruption, serotonin decreased local perfusion in several brain areas supplied by blood from the internal carotid artery [58], whereas local perfusion was increased in areas not supplied by the internal carotid artery [58,59]. Furthermore, elevated levels of histamine increased BBB permeability and reduced cerebral blood flow in rodent models [60]. Thus, serotonin and histamine stored and released by platelets play a role in modulating cerebral perfusion, which is likely of relevance, since specifically cerebrovascular damage and hypoperfusion has been associated with dementia (reviewed by Iadecola et al. [61]).

In summary, the secretion of stored growth factors by platelets such as VEGF increased BBB permeability by the expression of tight junctions, whereas PDGFB promote vascular integrity by supporting endothelial cell proliferation and recruiting pericytes. The release of serotonin and histamine by platelets could affect cerebral perfusion. These findings suggest a fundamental role of platelets balancing vascular integrity, BBB permeability and cerebral perfusion in MetS.

### 3.2. Adult Neurogenesis

Neurogenesis, the generation of new neurons from neuronal precursor cells, occurs in two main brain regions in adulthood: the subgranular zone of the hippocampal dentate gyrus and in the subventricular zone (SVZ). These findings also indicate that adult neurogenesis can be influenced by MetS [62,63]. Platelet microvesicles contain a large range of bioactive molecules, including cytokines and chemokines such as PF4 [13,14,15,64]. PF4 inhibits endothelial cell migration [65], recruits monocytes to the endothelium [66] and promotes neuronal differentiation in neural precursor cells [67]. Two recent reviews underlined the potential of microvesicles to regulate neural precursor cells [7,68], and moreover reported that administration of microvesicles increases the number of newly formed neuroblasts and promotes neurovascular remodeling after stroke [69]. The brain’s health and function profoundly depends on an adequate cerebrovasculature, and specifically during adults neurogenesis an angiogenic niche is formed in the SVZ and dentate gyrus of the hippocampus [70]. These findings indicate that angiogenesis and neurogenesis are tightly coupled in adult neurogenesis [70]. Thereby, platelets are interesting anucleate cells to consider in relation to neurogenesis in the dentate gyrus [67] and in the SVZ [71,72,73,74]. Some growth factors, such as VEGF [75], IGF-1 [76], FGF-2 [77,78], and thrombospondin-1 [79], which can be present in α-granules, induce angiogenesis and hippocampal neurogenesis. In addition, platelets contain other neurogenesis-promoting molecules in dense granules such as serotonin [80] and histamine [81].

Additionally to activated platelets, the overlapping features of MetS, hyperlipidemia, hyperglycemia and low-grade systemic inflammation can affect neurogenesis, as, for example, hippocampal neurogenesis can be disrupted by an excessive level of pro-inflammatory cytokines [82], and in zebrafish and in the SVZ of rats it has been reported that hyperglycemia impaired neurogenesis [83,84]. Bracke et al. found a reduced level of immature neurons in the hippocampus of a leptin-deficient obese mouse model for T2DM [62], whereas upon high fat diet (HFD)-feeding, female mice showed an increased level of neurogenesis in the SVZ [63]. Peroxidized lipid accumulations in the hippocampus and impaired hippocampal neurogenesis were found in young hyperlipidemic mice [85].

Regarding the strengths of the regulatory functions of platelets, particularly their abundant neurogenesis-promoting molecules and release upon activation in MetS, more research is needed to elucidate the influence of activated platelets in neurogenesis in MetS.

### 3.3. Neuroinflammation and Glial Cells

Widely studied in translational models, metabolic overload triggers hyperglycemia, hyperlipidemia and low-grade systemic inflammation and can induce neuroinflammation, specifically by inducing astrocytosis and activation of microglia [86,87,88]. Activated platelets can secrete various cytokines (e.g., interleukin-1, soluble cluster of differentiation 40 ligand (sCD40L) and chemokines (e.g., PF4, chemokine ligand-1, 5 (CCL5), 7 and 8) from α-granules, which provide pro-inflammatory signals organizing (vascular) leukocyte recruitment and tissue repair (for reviews, see [89,90]). For instance, the platelet-derived cytokine, sCD40L, induced neuroinflammation and neuronal death in the hippocampus and cortex [91]. In more detail, activation of platelets via ADP induced sCD40L release and the activation of astrocytes and microglia in hypertensive rats [91]. Notably, platelet-rich plasma induced prominent activation of astrocytes and microglia and a release of the pro-inflammatory cytokine TNF-α in rats [91]. When these rats were injected with a neutralizing antibody to sCD40L or a purinergic receptor (P2Y) G-protein coupled 12 (P2Y12) antagonist, which inhibits ADP-regulated platelet aggregation (clopidogrel), the sCD40L-induced neuroinflammation and TNF-α release were reversed [91]. In agreement with this, increased sCD40L levels have been found in patients with hypertension [92], T2DM [93,94], obesity [95] and MetS [94,96,97,98,99]. These results suggest that platelet sCD40L is a critical mediator of astrocyte and microglia activation, neuroinflammation, and in particular links platelet-derived sCD40L with neuroinflammatory responses in the brain in MetS. Additionally, excessive CCL5 expression can result in high levels of neuroinflammation via the activation of microglia, which can evolve into neurodegenerative processes (for review [100]). Moreover, neuroinflammatory processes can induce activated platelet accumulation in brain parenchyma [101], and it was shown that astroglial and neuronal lipid rafts induced platelet degranulation and secretion of neurotransmitter, serotonin [101,102] and pro-inflammatory factors such as platelet-activating factor (PAF) [101,102,103]. In detail, regulatory serotonin is released by activated platelets from dense granules [104], while PAF is mostly expressed on the surface of platelet-derived microvesicles [105] and exerts a pro-inflammatory role [106]. Notably, microvesicles have the potential to cross the BBB; interestingly, this potential movement is bidirectional [10]. These findings suggest that platelets have a role in the regulation of neuroinflammation. As a consequence, chemokines and cytokines released by platelets have crucial roles in the regulation of pro-inflammatory processes at the BBB, inducing neuroinflammatory processes and, when present in excessive amounts, even leading to neurodegeneration. 

In parallel, obesity and MetS are associated with a reduction in myelin and microstructural changes in white matter [107,108] and with an increased level of white matter hyperintensities in the brain [109,110]. Additionally, metabolic dysfunction induces oligodendrocyte loss [111] and structural defects in myelin sheaths in the central nervous system [112]. PDGF or PAF could affect myelinization; for instance, PDGF signalling is essential to oligodendrocyte differentiation and myelination in the central nervous system [113]. PAF is produced by a variety of cells, but especially those involved in host defence, such as platelets, endothelial cells, neutrophils, monocytes, and macrophages. Thus, PAF can activate platelets by binding to their G-protein-coupled PAF receptor and upon activation by other factors (e.g., thrombi), platelets synthesize and secrete PAF [114]. An in vitro experiment showed that administration of the biologically active lipid metabolite, PAF C-16, resulted in a significant level of apoptosis in cultured oligodendrocytes and astrocytes via activation of the caspase-3 pathway [115]. Next to this, PAF functions as a key messenger in neurone-microglial interactions [115].

All in all, sCD40L can induce neuroinflammation by astrocytosis and activation of microglia, whereas PDGF and PAF modulate myelinization via apoptosis and oligodendrocyte differentiation. Thus, platelet-derived compounds such as cytokines, chemokines and growth factors (e.g., sCD40L, PDGF and PAF) affect neuroinflammation and myelinization. These findings highlight the crucial role of platelets in neurovascular processes and stress the potential detrimental effects of hyperactivated platelets during MetS.

## 4. Nutritional Compounds in Platelet Activation

**Figure 3:**
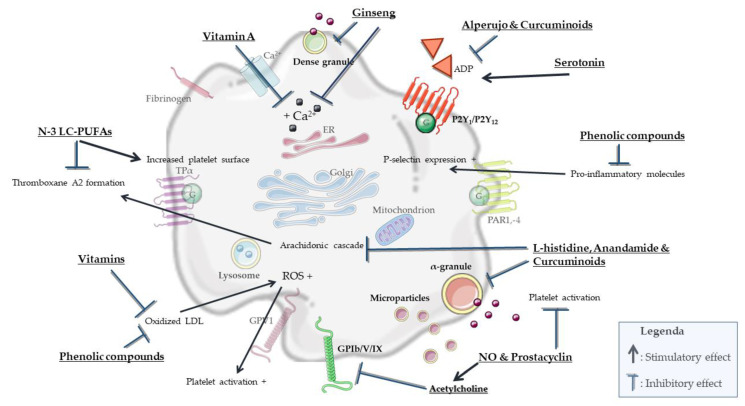
Schematic representation of mechanisms whereby nutritional compounds can affect platelet function.

Dietary bioactive compounds (e.g., n-3 LC-PUFAs, vitamins and polyphenols) can significantly contribute to normal platelet function (Figure 3), and thereby play a vital role in maintaining cardiovascular health in MetS.

### 4.1. LC-PUFAs and Derivatives

The most convincing evidence for dietary compounds that reduce platelet activation has been found in the consumption of LC-PUFAs [116,117,118]. Byelashov et al. reported that docosahexaenoic acid (DHA) reduced blood platelet aggregation [119]. A study by Vericel et al. examined the effects of 400 mg/day of DHA intake for 2 weeks on collagen-induced blood platelet aggregation and lipid peroxidation in 11 post-menopausal women with T2DM [120]. They found that daily DHA supplementation in moderate amounts reduced platelet aggregation by about 47% and lowered thromboxane A2 biosynthesis by about 35% in platelets [120]. However, the underlying biochemical mechanisms mediating these beneficial effects are only partly known. Adili et al. reviewed the three main n-3 LC-PUFAs obtained from food: eicosapentaenoic acid (EPA), DHA and alpha-linolenic acid, which are commonly found in fatty fish (EPA and DHA) and in vegetable oils and nuts (especially walnuts), flax seeds and flaxseed oil, leafy vegetables, and some animal fats, especially from grass-fed animals (alpha-linolenic acid) [121]. They reported that n-3 LC-PUFAs acted on the platelet membrane to reduce platelet aggregation and thromboxane release by acting on cyclooxygenases (COX)-1 and on 12- lipoxygenases (LOX) [121]. COX-1 and 12-LOX are two important oxygenases involved in metabolizing PUFAs into oxylipins [36,122]. Oxylipins are formed from fatty acids by pathways involving dioxygen-dependent oxidation, and often activate platelets [36,122]. Interestingly, the n-3 PUFA, docosapentaenoic acid (DPA) and derived oxylipins inhibited platelet reactivity in mice. The inhibitory effect on platelet activation of DPA-derived oxylipins was found to be mediated through the activation of the peroxisome proliferator activator receptor (PPAR) -α [123], and moreover could be regulated via COX-1 and 12-LOX [121].

Furthermore, n-3 LC-PUFAs increased the total platelet surface charge and thereby attenuated platelet activation, even among patients taking aspirin or aspirin plus clopidogrel [124]. Moreover, n-3 LC-PUFAs can be incorporated into platelet membrane phospholipids, leading to a concomitant reduction in n-6 LC-PUFAs along with an increase in EPA and DHA [125]. EPA can then compete with arachidonic acid and inhibit the COX-1 pathway. Finally, n-3 PUFAs DHA and EPA can be converted, via nonoxidative pathways, into endocannabinoiods such as docosahexaenoyl ethanolamide (DHEA) and eicosapentaenoyl ethanolamide (EPEA), through N-acyl ethanolamine synthesis [126,127]. The endocannabinoid anandamide (arachidonoylethanolamide) is known to inhibit platelet aggregation and α-granule release by collagen, collagen-derived peptide CRP-XL, ADP, arachidonic acid cascade and subsequently the formation of thromboxane A2 [128] (Figure 1). Anandamide-treated platelets exhibited reduced spreading on immobilized fibrinogen, had a decreased capacity for binding fibrinogen in solution, and showed perturbed platelet aggregate formation under flow over collagen [128]. Additionally, De Angelis et al. showed that an endocannabinoid receptor agonist reduced platelet activation and aggregate formation both in vitro and ex vivo [128]. In contrast, high levels of the endocannabinoids 2-arachidonoylglycerol and virodhamine stimulated platelet activation [129,130], which was explained by the conversion of these endocannabinoids to arachidonic acid and subsequently thromboxane A2, not by the activation of endocannabinoid receptors [131]. Moreover, endocannabinoids can be metabolized by eicosanoid synthesizing enzymes from the COX, LOX and cytochrome P450 oxygenase pathways to generate complex lipid metabolites with distinct biological functions [132]. Endocannabinoid epoxides may regulate plated function, and in particular, epoxyeicosatetraenoic acid-ethanolamide (EEQ-EA) was shown to inhibit platelet aggregation [126]. Finally, a recent study showed that n-3 PUFA-derived endocannabinoid epoxides, epoxyeicosatetraenoic acid-ethanolamide and epoxydocosapentaenoic acid-ethanolamide, derived from DHA and EPA, respectively, exert anti-inflammatory and vasodilatory effects, and reciprocally modulate platelet aggregation [126,133].

### 4.2. Vitamins and Polyphenols

Extensively reviewed by Kobzar et al., some vitamins, including vitamin A, D, B_6_ and C, can act as inhibitors of platelet activation by inhibiting biochemical pathways or preventing damage to vessel walls [134]. Particularly in combination, vitamins may act synergistically to enhance the effects of endogenous anti-platelet compounds, such as prostacyclin or nitric oxide [134]. The vitamins with the most potent antiplatelet activity are vitamins A and D [134]. Specifically, vitamin A inhibits intracellular calcium release and platelet inhibition via the binding of an intracellular retinoid X receptor with a G-coupled protein [134,135], and vitamin D inhibits platelet aggregation by modulating endothelial cells [134]. Fruits are a source of vitamins and moreover polyphenols, which together may induce antioxidant effects and reduce platelet activation [136]. For instance, high concentrations of phenolic compounds can be found in fresh berries and berry extracts, i.e., phenolic acid and flavonoids [136]. Specifically in combination, vitamins and polyphenols can inhibit platelet activation via antioxidant effects, modulation of endothelial cells or inhibiting intracellular calcium release [134,136].

### 4.3. Amines and Amino Acids

The dense granules of platelets contain amines such as serotonin, histamine, gamma-aminobutyric acid (GABA) and glutamate. Plausibly, these amines, also functional as neurotransmitters, could affect microglia upon release in cases of increased BBB permeability. Dietary intake of the precursors of these amines could affect platelet aggregation and subsequently even neurological function. In more detail, serotonin is synthesized from tryptophan to 5-hydroxy-tryptophan by either tryptophan hydroxylase-1 (brain, 5%) or -2 (periphery, mainly enterochromaffin cells in the gastrointestinal tract 95%) [137]. Diets enriched with tryptophan (4 and 10 g/kg) enhanced ADP-induced platelet aggregation, most likely via the (increased) synthesis of serotonin, and may thereby contribute to atherosclerotic risk [138]. In addition, in hypercholesteremic rats, excessive dietary tryptophan increased plasma lipid peroxidation and macrophage cholesterol esterification [139]. These effects were associated with the increase in serotonin levels, as serotonin enhanced LDL peroxidation, whereas tryptophan had no effect on LDL peroxidation [139]. Moreover, serotonin is a weak platelet agonist and dose-dependently enhances platelet activation induced by ADP and thrombin [140]. Thus, excessive levels of tryptophan can affect peripheral serotonin levels and thereby by ADP-induced platelet aggregation and LDL peroxidation.

The amino acid l-histidine can be condensed with decarboxylation to form histamine [141]. In a seven day intervention, participants either received placebo or 1-histidine (3 g/day), and in this study it was shown that l-histidine effectively inhibited spontaneous platelet aggregation [142]. This effect was probably mediated by arachidonic acid metabolites [142]. Thus, dietary intake of the precursor of histamine, l-histidine, could inhibit platelet aggregation. Additionally, dietary histidine intake was negatively associated with pro-inflammatory cytokines such as tumor necrosis factor alpha, interleukin-1 and -6 and inflammation biomarker c-reactive protein, and thereby might reduce inflammatory processes, specifically individuals with MetS [143,144,145].

Lastly, human platelets express glutamate receptors and have a high affinity for the uptake of glutamate [8,143]. Interestingly, tryptophan, sodium glutamate and histamine are available in tomatoes, and a study by Yamamoto et al. showed an effect on platelets activity independent of coagulation, and dependent on tomato varieties [146]. The platelet aggregation was significantly inhibited at all stages of ripening, but mostly at the green and pink phase compared to the mature and over-mature phase [146]. An interesting amine to examine in this particular process would be GABA, as it can be synthesized from sodium glutamate, tomatoes contain a relatively high level of GABA and platelets contain GABA [8,144]. In particular, the GABA levels increase from flowering to the mature green stage and then rapidly decrease during the ripening stage [145]. During the green stage GABA constitutes up to 50% of the free amino acids in tomatoes [145]. In addition, consumption of tomato products attenuates postprandial oxidative stress induced by lipemia and associated inflammatory response [147].

All in all, dense granules of platelets carry amines such as serotonin, histamine, GABA and glutamate, and particularly the dietary intake of their precursors could affect platelet aggregation, and some of the amines can affect ADP-induced platelet aggregation, inflammatory response and LDL peroxidation. Future research should take into account the role of precursors of amines in platelet activation and aggregation in MetS.

### 4.4. Extracts of Fruits and Plants

Other food products, extracts or nutrients which might exert antiplatelet effects include, for example, olive oils, alperujo, ginseng, curcuminoids and garlic. After the extraction of oil from the olive, many phenolic compounds remain in the by-product alperujo. De Roos et al., showed that in vitro ADP- and TRAP-induced platelet activation was significantly decreased by alperujo extract (40 mg/L), and in particular, alperujo extract regulated proteins involved in processes such as the regulation of platelet structure and aggregation, coagulation, apoptosis, and signalling by integrin αIIb/β3 [148]. Elsewhere, it has been found that oral supplementation for one year with extra virgin olive oil enriched with vitamins (K1, D3 and B6) reduced blood platelet aggregation stimulated by ADP [149,150]. Notably, natural olive phenols had an inhibitory effect on human platelet aggregation, and in particular, hydroxytyrosol is one of the major phenolic compounds in olive oil [151]. Ginseng has been used as a traditional preventive and therapeutic herbal medicine against several diseases, especially cardiovascular disease. Broad-spectrum antiplatelet effects of ginsenosides could be attributed to their ability to attenuate internal calcium mobilization and granule secretion [152]. Curcuminoids, extracted from Curcuma longa plants, significantly inhibited platelet aggregation induced by modulating ADP and arachidonic acid [153]. Finally, aged garlic extract inhibits platelet aggregation by increasing cyclic nucleotides, inhibiting fibrinogen binding, attenuating platelet shape changes and changing the functional properties of platelets to respond to collagen [154,155].

All in all, several nutritional compounds (Figure 3) have shown the ability to attenuate platelet activation such as n-3 LC-PUFAs, vitamins, berries, l-histidine, tomatoes, olive oils, ginseng, curcuminoids and aged garlic extract. These products and nutrients can often affect platelet activation via combined effects such as antioxidant activity, increasing the total platelet surface, affecting the arachidonic cascade, inhibiting fibrinogen binding and increasing levels of cyclic nucleotides. Exact mechanisms for many of these specific nutrients are not known in detail and are hard to identify as a single mechanism, and the strongest and most effective antiplatelet effects appear to be provoked by combining nutrients.

## 5. Dietary Compounds and Platelet Activation in MetS

In this section, we aim to integrate dietary intervention studies which examined the effect of dietary bioactive compounds on platelet function and neurovascular processes in MetS. Only limited studies were found examining neurovascular parameters. Ras et al. examined the effect of an eight-week supplementation with a flavonoid source, grape seed extract, in individuals with hypertension (stage 1), and found no significant effects in platelet aggregation [156]. However, a study by Thompson et al. revealed that 28 days (320 mg/d) of supplementation with anthocyanins, a subclass of the polyphenol family, reduced ADP-induced platelet activation, platelet aggregate formation and platelet endothelial cell adhesion in individuals with overweight or obesity (BMI > 25.0 kg/m^2^) [157]. Interestingly, a high-fat meal can induce platelet aggregation, which was shown to be attenuated when the meal was enriched with a source of antioxidants, such as vegetables and vitamins (tomatoes, vitamin C, vitamin E, β-carotene (provitamin A)) [158]. In particular, the Mediterranean diet is known for its high content of n-3 LC-PUFAs, antioxidants and phenolic compounds. Recently, it was shown that that the incorporation of boiled wild plants in a mixed meal can attenuate post-meal increases in PAF-induced platelet aggregation in metabolic syndrome patients. Furthermore, components of the Mediterranean diet can favorably modulate the pro-inflammatory actions of PAF and modulate its metabolism [159]. Individuals with MetS adhering to the Mediterranean-style diet improved in blood pressure and platelet aggregation in response to L-arginine injection after 2 years [160]. These individuals consumed more foods rich in polyunsaturated fat and had a lower ratio of n-6 to n-3 LC-PUFAs, and their fruit, vegetable, and nut intake and olive oil consumption were also significantly higher [160]. A multidisciplinary approach consisting of diet, exercise, behavioural and nutritional counselling in obese women significantly reduced the platelet aggregation in response to L-arginine injection [161]. Interestingly, the diet used in this multidisciplinary approach was very similar to the Mediterranean-style Step I diet [161]. These observations merit further human intervention studies to examine the effects of dietary factors on platelet activation in MetS and specifically on neurovascular parameters.

## 6. Conclusions

**Figure 4:**
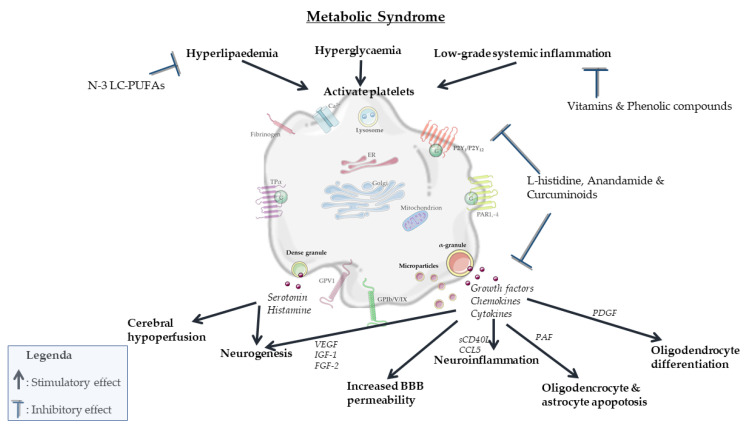
Graphical summary of dietary bioactive compounds on platelet function and neurovascular processes in MetS.

In summary, partly overlapping processes involved in MetS can activate platelets mainly via intracellular changes in osmolality, calcium concentration, membrane charge and oxidation and glycosylation of LDL (Figure 2). In turn, activated platelets can mainly stimulate neurodegenerative processes associated with MetS by modulating vascular integrity, BBB permeability, neurogenesis, myelinization and neuroinflammation. Specific nutritional components, including n-3 LC-PUFAs, antioxidants and phenolic compounds, attenuate both platelet activation (Figure 3) and pathological processes in the vasculature and brain parenchyma (Figure 4). However, a direct causal relationship between these two effects remains to be more firmly established. In particular, diets in which n-3 LC-PUFAs, antioxidants and phenolic compounds are present in combination, such as the Mediterranean diet, attenuate platelet activation and aggregation in MetS. However, the effects of these compounds and diets specifically in association with both platelet activation and neurovascular parameters are underexamined in MetS. Therefore, future research should elucidate the exact role and biological mechanisms of platelets in pathological neurodegenerative processes associated with MetS, particularly accelerated neurological aging, vascular dementia and AD. When such modifiable connections can be demonstrated, this may support the development of novel preventive or therapeutic intervention strategies.

This review underlines that activated platelets can have a significant contribution in neurovascular deterioration, and that specific endogenous and nutritional compounds can modulate these processes by attenuating platelet activation. It also restresses that a balanced diet is of importance to prevent or treat metabolic and neurological pathologies, and that it is plausible that the smallest anucleate cells of the body play roles in this process.
